# eCAMBer: efficient support for large-scale comparative analysis of multiple bacterial strains

**DOI:** 10.1186/1471-2105-15-65

**Published:** 2014-03-05

**Authors:** Michal Wozniak, Limsoon Wong, Jerzy Tiuryn

**Affiliations:** 1 Faculty of Mathematics, Informatics and Mechanics, University of Warsaw, Warsaw, Poland; 2 School of Computing, National University of Singapore, Singapore, Singapore

**Keywords:** Comparative genomics, Bacteria, Genome annotation

## Abstract

**Background:**

Inconsistencies are often observed in the genome annotations of bacterial strains. Moreover, these inconsistencies are often not reflected by sequence discrepancies, but are caused by wrongly annotated gene starts as well as mis-identified gene presence. Thus, tools are needed for improving annotation consistency and accuracy among sets of bacterial strain genomes.

**Results:**

We have developed *eCAMBer*, a tool for efficiently supporting comparative analysis of multiple bacterial strains within the same species. eCAMBer is a highly optimized revision of our earlier tool, *CAMBer*, scaling it up for significantly larger datasets comprising hundreds of bacterial strains. eCAMBer works in two phases. First, it transfers gene annotations among all considered bacterial strains. In this phase, it also identifies homologous gene families and annotation inconsistencies. Second, eCAMBer, tries to improve the quality of annotations by resolving the gene start inconsistencies and filtering out gene families arising from annotation errors propagated in the previous phase.

**Conculsions:**

eCAMBer efficiently identifies and resolves annotation inconsistencies among closely related bacterial genomes. It outperforms other competing tools both in terms of running time and accuracy of produced annotations. Software, user manual, and case study results are available at the project website: http://bioputer.mimuw.edu.pl/ecamber.

## Background

The number of bacterial genome sequences available in public databases is growing rapidly, due to advances in high-throughput sequencing technologies [1]. For example, from June 8, 2011 to February 12, 2014, the total number of whole-genome sequences available in the PATRIC database grew from 3303 to 14114 [2]. By December 16, 2013, there were 1452 whole-genome sequences of *Escherischia coli* and 435 whole-genome sequences of *Salmonella enterica* strains available in the database.

Larger datasets of bacterial genome sequences enable new interesting comparative genome analysis [3-7]. However, it has been shown that a wide range of comparative analyses (such as identification of overlapping genes and estimation of core genome size) may be complicated or biased due to the common inconsistencies in genome annotations among closely related bacterial strains [8-13].

The observed inconsistencies are mostly of two types: mis-identification of gene presence (false positive and false negative predictions are possible) and inconsistent gene starts (or TIS — translation initiation sites). It has also been argued that most of these inconsistencies are not reflected by sequence discrepancies, but arise as a result of different annotation methodologies applied by different laboratories [10,14]. In fact, has been shown that using the same tool to annotate a set of bacterial genomes increases annotation consistency [10]. However, as we will observe later in section “Annotation consistency”, these annotation inconsistencies among closely related genomes can even arise from annotations produced by the same annotation tool or made by the same laboratory.

There is also an interesting question regarding TIS inconsistencies: *can a bacterial gene have multiple TISs?* For example, it has been recently estimated, based on an experimental study, that as many as 26.5% of genes in *E. coli* may have multiple transcription start sites [15]; that may also suggest multiple TISs. Nevertheless, according to our knowledge, multiple real TISs *in bacteria* is not a confirmed phenomenon yet. It should also be noted that there is only one TIS per gene in manually curated annotations. Thus, in this study, we assume that each gene has only one correct TIS.

Interestingly, the presence of annotation inconsistencies is an expected phenomenon when single-genome prediction tools are applied independently. For example, suppose we annotate independently *k*=20 genomes, and assume that the missing gene error rate is *ε*=0.035, which is the corresponding Prodigal [16] error rate estimated on the *E. coli* dataset. Then, since 1−(1−*ε*)^*k*^=0.51, about 51% of core gene families would have at least one missing gene annotation.

A promising idea to improve annotation accuracy by combining outputs of several single-genome annotation tools has been explored with a few proposed approaches [17-20]. However, these meta-approaches can be viewed as single-genome annotation tools.

Recently, it has also been proposed that the accuracy of single-genome annotation tools can improved by comparative annotation among multiple genomes [21]. However, even though there are many annotation tools dedicated to a single-genome, there are relatively few tools supporting comparative annotation and analysis of multiple bacterial genomes [21]. Hence, there is a need to develop more tools to improve consistency of genome annotations across multiple bacterial strains.

Mugsy-Annotator is a tool which may assist in the curation of annotations of multiple bacterial genomes by identifying annotation inconsistencies [22]. First, this tool computes whole-genome multiple alignment by employing Mugsy [23]. Then, based on annotated gene coordinates mapped on genomes in the multiple-genome alignment, Mugsy-Annotator identifies orthologous gene families, annotation inconsistencies and proposes changes to the input annotations. Notably, Mugsy-Annotator does not make any assumption about the reference strain. However, it suffers from the quadratic time complexity with respect to the number of strains, since in the first step it employs Mugsy to compute pairwise all-against-all alignments of whole genomes.

Recently, two new mojority voting-like approaches have been proposed to improve annotation accuracy and consistency among multiple genomes: ORFcor [24] and GMV [25]. However, ORFcor requires a set of ortholog gene families to be supplied as the input, and GMV is embedded within a pipeline which starts from input genome sequences and genome annotations generated by Prodigal. It should also be noted, that since the GMV pipeline uses BLAST in the all-against-all manner it has quadratic time complexity with respect to the number of strains.

In our previous work, we developed CAMBer [11], a tool conceptually similar to Mugsy-Annotator and the GMV pipeline. It supports comparative analysis of multiple bacterial strains. CAMBer unifies input gene annotations by homologous gene transfer among all strains. Then, based on acceptable BLAST hits, it identifies orthologous gene families. During this procedure annotation inconsistencies are identified. Similarly, as in Mugsy-Annotator and the GMV pipeline, it does not make any assumption about the reference strain, and it has quadratic time complexity in the number of strains. This property makes both tools weakly scalable to large datasets.

Another notable tool which employs the idea of comparing gene annotations among closely related genomes is GenePRIMP [26]. This tool identifies and reports gene annotation anomalies based on protein BLAST queries run against the NCBI nr database. These reports are helpful for manual curation of genome annotations. A similar feature has also been implemented in CAMBerVis [27] — our previously published tool for visualization and analysis of annotation inconsistencies.

In this work, we present a new version of CAMBer, which we call *eCAMBer* (efficient CAMBer). It also aims to identify annotation inconsistencies and orthologous gene families. However, unlike Mugsy-Annotator and CAMBer, it has significantly better running time by taking advantage of working with highly similar genome sequences. A dramatic speed up offered by eCAMBer can be seen when working with a large number of bacterial strains. The running time is reduced (for 41 strains of *E. coli*) from 2 days, in the case of CAMBer, to less than half an hour, in the case of eCAMBer. Furthermore, eCAMBer tries to resolve annotation inconsistencies in order to produce more accurate annotations. For this purpose, it implements a majority voting-like approach for selecting the most reliable TISs and implements a procedure for identification and removal of gene families which are likely to be propagated annotation errors.

The concept of annotation may refer to many different aspects of attaching biological information to genome sequences, such as: identifying of gene locations, assigning functions to genes or assigning network context to gene products [14,28,29]. In this work we focus on identifying locations of protein-coding genes. We use the term *gene annotation* (or *ORF annotation*) to refer to genome coordinates of a protein-coding gene from its translation initiation site TIS (alternatively called *gene start*) to the nearest stop codon (alternatively called *gene end*). Note that each ORF annotation is unambiguously determined by specifying strand and position of its start codon. Thus, we can use the term *TIS annotation* as a synonym to ORF annotation. We will be using this when multiple ORF annotations share the same stop codon.

## Methods

**Figure 1:**
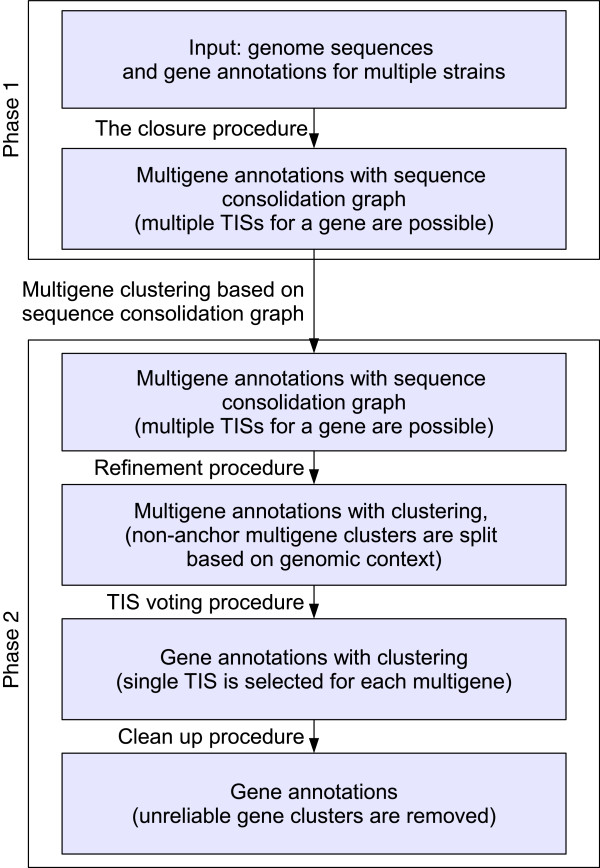
**Schematic view of subsequent procedures in eCAMBer.** Boxes of the chart represent the subsequent sets of annotations. Edges indicate application of eCAMBer procedures to process these annotations. We call a set of ORF annotations, *multigene annotations*, if multiple ORF annotations may share the same stop codon, indicating possible starts of translation (TISs). We use a notion of a *multigene* to represent multiple ORF annotations sharing the same stop codon.

eCAMBer requires as its input a set of genome sequences and annotations for multiple bacterial genomes. It should be noted, however, that eCAMBer supports automatic download of bacterial annotations from the PATRIC [2] database and, as an option, it allows the use of Prodigal to generate the input annotations. It works in two phases. In the first phase it uses BLAST+ [30] to transfer each gene annotation among multiple strains. Based on the results of this procedure, homologous multigene clusters are identified. In the second phase eCAMBer applies subsequently the procedures for refinement, TIS voting and clean up. Figure 1 presents a schematic view of these subsequent procedures of eCAMBer.

The main improvements in eCAMBer as compared to CAMBer [11] are: 

•Significant speed up of the *closure procedure* for unifying genome annotations among bacterial strains;

•Modified *refinement procedure* for splitting homologous gene families into orthologous gene clusters;

•New *TIS voting procedure* for selecting the most reliable TIS;

•New *clean up procedure* for removal of gene clusters that are likely to be gene annotation errors propagated during the *closure procedure*.

Here, we describe the details of the above listed procedures. The default values for parameters introduced below were chosen arbitrarily. However, based on our experiments, the program is robust for other choices of the parameters from a reasonable spectrum. eCAMBer allows users to specify values of all the parameters.

### The closure procedure

The closure procedure is the first step of eCAMBer. The input consists of genome sequences and genome annotations for a set of closely related bacterial strains. In this procedure gene annotations are iteratively transferred among the set of considered strains, until no new ORFs (open reading frames) are identified.More precisely, a gene annotation is transferred to a new location if its BLAST hit extended to the nearest in-frame stop codon is *acceptable*. Analogous to CAMBer, a BLAST hit extension to the nearest stop codon is *acceptable* if it satisfies the following conditions: 

•The hit has one of the appropriate start codons: ATG, GTG, TTG, or the same start codon as in the query sequence;

•The hit has its beginning aligned with the beginning of the query sequence;

•The BLAST e-value score is below a given threshold *e*_*t*_ (in the default setting *e*_*t*_=10^−10^);

•The ratio of the length of the extended hit to the query length is less than 1+*p*_*t*_ and greater than 1−*p*_*t*_, where *p*_*t*_ is a given threshold (in the default setting *p*_*t*_=0.2);

•The percentage of identity of the hit (calculated as the number of identities divided by the query sequence length, times 100) is above a length-dependent threshold given by the adaptation of the HSSP curve introduced in our previous work [11], defined by the parameter *n*_*t*_ (in the default setting *n*_*t*_=60.5).

In this procedure eCAMBer, unlike CAMBer, takes advantage of working with closely related genomes. In contrast to the old approach, in each iteration, instead of using each ORF sequence as a query, it first identifies groups of ORFs with exactly identical sequences. This approach avoids use of the same ORF sequence multiple times as a BLAST query.

The pseudocode for the closure procedure implemented in eCAMber is given in Algorithm ??, which we now describe in more details. First, we start with the set of input annotations $A_{s}^{0}$, for each strain *s* in the set of considered strains *S*. Each ORF annotation (or simply ORF) is defined by a tuple *(start, end, strand, contig, strain)*. Then, in *i*th iteration we compute the set of BLAST queries *Q*^*i*^ as the set of distinct ORF sequences among all strains, which have not been used as BLAST queries yet. Next, we calculate in parallel, for each strain, BLAST results for all sequence queries in *Q*^*i*^. For each strain *s*∈*S*, all acceptable BLAST hit extensions $H_{s}^{i}$ are added to the strain annotations, defining $A_{s}^{i+1}\leftarrow A_{s}^{i}\cup H_{s}^{i}$. Next, the set of newly identified sequences across all genomes *H*^*i*^ is computed, which is then used to update the set of BLAST queries for the next iteration *Q*^*i*+1^←*H*^*i*^∖*D*^*i*^, where *D*^*i*^ denotes the set of all distinct sequences before the *i*th annotation. The procedure stops when no new ORF sequences are identified, hence *Q*^*i*^=*∅*. For each strain *s*∈*S*, we denote by $A_{s}^{c}$ the set of annotations produced by the closure procedure above. We further denote by *A*^*c*^ the set of all ORFs produced by the closure procedure. 

Here, we also recall the notion of a *multigene*, introduced in our previous work [11], to account for the situation when multiple ORFs share the same stop codon in the annotations produced during the *closure procedure*. These ORFs are called multigene elements and represent putative gene translation units. Each *multigene* is represented by a tuple (*end*,*strand*,*contig*,*strain*,*elts*), where *elts* is the set of ORFs constituting the multigene. Also, for each strain *s*∈*S*, we denote by $M_{s}^{c}$ the set of multigenes resulting from the closure procedure.

**Figure 2:**
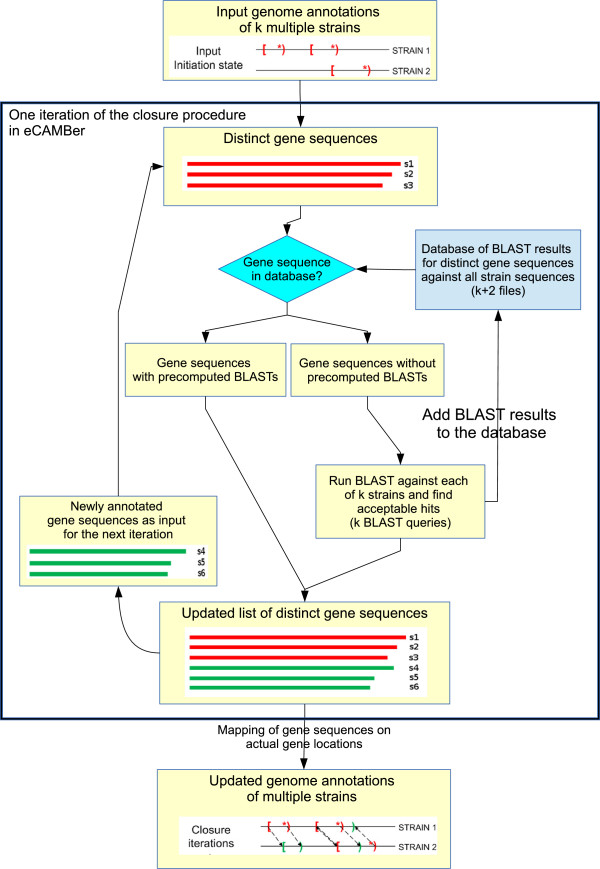
**Schematic view of the closure procedure in eCAMBer.** Schematic view of the closure procedure in eCAMBer. Annotations with multiple ORF annotations sharing the same stop codon may be produced. This situation gives rise to the notion of a *multigene*, which represents the set of ORFs sharing the same stop codon. These multigene elements correspond to putative gene translation units.

Figure 2 presents a schematic view of the implementation of the closure procedure in eCAMBer.

The careful reader may also notice two important differences between the closure procedure in CAMBer and eCAMBer. In particular, eCAMBer uses unique ORF sequences, rather than ORF annotations, as queries against all strain genomes and, thus, does not repeat a BLAST query when the same ORF sequence corresponds to multiple ORF annotations. In contrast, firstly, CAMBer uses all ORF sequences as queries and, thus, may repeat a query BLAST several times. Secondly, CAMBer BLASTs a query against all strains’ genomes except the strain from which the query is taken. The second difference may potentially lead to different outcomes generated by these two approaches.

**Figure 3:**
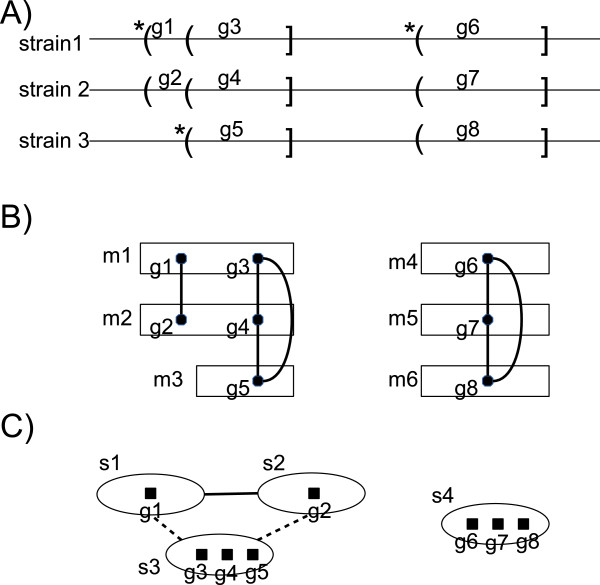
**Number of genes vs. number of distinct gene sequences.** Comparison of the number of distinct gene sequences to the total number of genes in original annotations of 569 strains of *E. coli*. Strains were included cumulatively in the order of increasing genome sizes. In the figure the x-axis corresponds to the number of strains included.

Since BLAST computations are the most time-consuming operation in each iteration of the *closure procedure*, we express the time complexity of one iteration of the *closure procedure* by the number of performed BLAST computations. Let *k*=|*S*| denote the number of considered strains and let $n={\text{max}}_{s\in S}|A_{s}^{i}|$ be the maximal number of gene annotations per strain, in iteration *i*. Let, *d*=|*D*^*i*^| denote the number of distinct gene sequences among all gene annotations in all considered strains. Then, the time complexity of one iteration of the closure procedure implemented in eCAMBer can be expressed as *O*(*d*·*k*), whereas it is *O*(*n*·*k*^2^) for CAMBer. Here, it should be noted that, potentially, if every annotated ORF sequence in *S* is different, then $\left|D^{i}|=\underset{s\in S}{\sum }|A_{s}^{i}|=O(n\cdot k\right)$. However, as our case study experiments show, *d* is usually much smaller than *n*·*k* (see Figure 3).

Importantly, the number of I/O operations per iteration is also significantly decreased, from *O*(*n*·*k*^2^) in CAMBer to *O*(*k*) in eCAMBer.

### Consolidation graphs

Having the closure procedure computed we represent its results in the form of graph structures, called *consolidation graphs*.

First, we introduce the conceptual representation, called the *ORF consolidation graph*. In this graph *G*_*O*_=(*V*_*O*_,*E*_*O*_), each node *o*∈*V*_*O*_ represents an ORF annotation in $A_{s}^{c}$, for some *s*∈*S*. There is an undirected edge {*o*_1_,*o*_2_}∈*E*_*O*_ between a pair of ORFs, if there is an acceptable BLAST hit from the sequence of *o*_1_ to *o*_2_ or from the sequence of *o*_2_ to *o*_1_. We additionally assume, that there are no self-edges, i. e. *o*_1_≠*o*_2_.

Second, we recall the definition of the *multigene consolidation graph*, introduced in our previous work [11]. In this graph *G*_*M*_=(*V*_*M*_,*E*_*M*_) each node *m*∈*V*_*M*_ represents a multigene in $M_{s}^{c}$, for some *s*∈*S*. There is an undirected edge {*m*_1_,*m*_2_}∈*E*_*M*_ between a pair of multigenes, if there is a pair of ORFs *o*_1_∈*elts*(*m*_1_) and *o*_2_∈*elts*(*m*_2_), such that there is an edge between them in the *ORF consolidation graph* (i.e., such that {*o*_1_,*o*_2_}∈*E*_*O*_).

Finally, we introduce the *sequence consolidation graph*, which is the structure used in the implementation of eCAMBer, as it is a compact representation of the information stored in the ORF consolidation graph and the multigene consolidation graph. In this graph *G*_*S*_=(*V*_*S*_,*E*_*S*_,*E*_*B*_), nodes represent distinct ORF sequences. There are two types of edges, *E*_*B*_ called *BLAST-hit edges*, and *E*_*S*_ called *shared-end edges*. There is an undirected *shared-end edge* {*x*,*y*}∈*E*_*S*_ between a pair of sequence nodes if there is a multigene having two elements with these sequences. There is an undirected *BLAST-hit edge* {*x*,*y*}∈*E*_*B*_ between a pair of sequence nodes if there is an acceptable BLAST hit from *x* to an ORF with sequence *y*, or if there is an acceptable BLAST from *y* to an ORF with sequence *x*.

**Figure 4:**
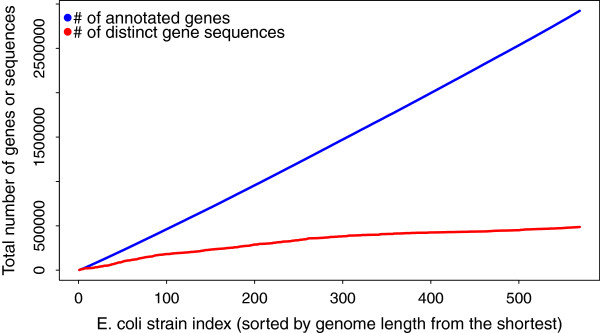
**Schematic view on the correspondence between different representations of the*closure procedure* results in the form of consolidation graphs.** Schematic view on the correspondence between different representations of the *closure procedure* results in the form of consolidation graphs; **A)** the genomes with marked ORF annotations. Round and square brackets represent the ORF start and stop codons, respectively. Round brackets with stars indicate original TIS annotations, whereas those without starts indicate the transferred TIS annotations; **B)** multigene representation of the annotations with the *ORF consolidation graph* edges shown between multigene elements, edges of the *multigene consolidation graph* are not shown for the readability; **C)** the *sequence consolidation graph* in which nodes correspond to the distinct ORF sequences, *shared-end* edges are drawn dashed, whereas *BLAST-hit* edges are drawn solid.

Figure 4 illustrates the correspondence between the ORF consolidation graph, sequence consolidation graph and the multigene consolidation graph.

### Homologous gene clusters

The second step of eCAMBer is to determine homologous gene families as connected components of the multigene consolidation graph *G*_*M*_. There is a natural one-to-one correspondence between the connected components of the multigene consolidation graph and the connected components of the sequence consolidation graph (the latter connected components are obtained by taking the union of *E*_*S*_ and *E*_*B*_). So, in eCAMBer, we do this using connected components of the sequence consolidation graph *G*_*S*_, because it tends to be smaller for closely related genomes. The obtained set of homologous gene families is represented as a set of disjoint *multigene clusters*, denoted by *C*_*M*_.

### Refinement procedure

The third step of eCAMBer is the *refinement procedure*. The goal of the *refinement procedure* is splitting the homologous gene families, represented by multigene clusters, to obtain anchors. We call a multigene cluster an *anchor*, if it includes at most one multigene for every strain. Analogously, we call a multigene cluster *non-anchor*, if there is a strain which includes at least two multigenes in the cluster. Multigenes in the same anchor are potentially orthologous to each other, whereas a non-anchor contains at least two multigenes that are non-orthologous. Following CAMBer, we use genomic context information to decompose non-anchors into smaller multigene clusters that can emerge as anchors, as described below.

The input for the refinement procedure consists of the set of multigene clusters *C*_*M*_, the sequence consolidation graph *G*_*M*_, and the multigene annotations $M_{s}^{c}$, for each strain *s*∈*S*. We start with classifying the set *C*_*M*_ of multigene clusters into two disjoint sets of anchors and non-anchors, denoted *C*_*A*_ and *C*_*N*_, respectively. We also sort all multigenes within strain contigs by positions of their stop codons. We reconstruct the subgraph of the multigene consolidation graph, called the *refinement graph*. In this graph *G*_*R*_=(*V*_*R*_,*E*_*R*_), nodes *V*_*R*_ are constituted by the subset of multigenes, which belong to non-anchor clusters. There is an edge {*m*_1_,*m*_2_}∈*E*_*R*_, between a pair of multigenes coming from different strains, if there is an edge {*m*_1_,*m*_2_}∈*E*_*M*_, and the two multigenes belong to the same multigene cluster. By $E_{R}^{\left\{s_{1},s_{2}\right\}}$ we denote the subset of edges between multigenes from a pair of strains *s*_1_ and *s*_2_. We omit details of the reconstruction of the refinement graph for brevity.

Then, for each unordered pair of strains {*s*_1_,*s*_2_} we perform the following procedure in parallel. First, for each multigene *m* we identify a pair of its nearest neighbours which belong to anchors with a multigene element present on the opposite strain. Such left and right neighbours of *m* are denoted as $l_{m}^{\left\{s_{1},s_{2}\right\}}$ and $r_{m}^{\left\{s_{1},s_{2}\right\}}$, respectively. Then, for each edge $\{m_{1},m_{2}\}\in E_{R}^{\left\{s_{1},s_{2}\right\}}$ we check whether it is *supported* in the sense that it satisfies one of the following conditions: (i) it connects multigenes belonging to a cluster, such that *m*_1_ and *m*_2_ are its only elements in strains *s*_1_ and *s*_2_; (ii) the corresponding pairs $\left(l_{m_{1}}^{\left\{s_{1},s_{2}\right\}},l_{m_{2}}^{\left\{s_{1},s_{2}\right\}}\right)$ and $\left(r_{m_{1}}^{\left\{s_{1},s_{2}\right\}},r_{m_{2}}^{\left\{s_{1},s_{2}\right\}}\right)$ belong to the same anchor; (iii) the corresponding pairs $\left(l_{m_{1}}^{\left\{s_{1},s_{2}\right\}},r_{m_{2}}^{\left\{s_{1},s_{2}\right\}}\right)$ and $\left(r_{m_{1}}^{\left\{s_{1},s_{2}\right\}},l_{m_{2}}^{\left\{s_{1},s_{2}\right\}}\right)$ belong to the same anchor. If any of the four neighbours does not exist we substitute it with a dummy node, which virtually belongs to any anchor.

Finally, we obtain the *refined graph*$G_{R}^{*}$ by removal of unsupported edges from *G*_*R*_. Then, the set of connected components *C*_*R*_ of $G_{R}^{*}$ defines the set of multigene clusters after the split. Finally, we update the set of multigene clusters as $C_{M}^{*}\leftarrow (C_{M}\setminus C_{N})\cup C_{R}$.

The careful reader may also notice the differences between the refinement procedures implemented in CAMBer and eCAMBer. First, the refinement procedure in CAMBer performs in iterations until no multigene clusters can be split. In eCAMBer the refinement procedure consists of only one iteration. However, since the input and output for the procedure are of the same type, it can be used multiple times, until no new clusters are split. Second, the condition for an edge to be supported in eCAMBer is more relaxed than that in CAMBer. Both approaches, for a pair of multigenes on different strains, identify pairs of their nearest left and right neighbour multigenes (belonging to anchor clusters with elements on both strains). However, CAMBer checks the actual presence of edges between the neighbours, whereas eCAMBer only checks if the identified neighbours match the same pair of clusters. This approach allows eCAMBer to avoid a costly reconstruction of the whole multigene consolidation graph.

### TIS voting procedure

The fourth step of eCAMBer is the *TIS voting procedure*. The goal of the TIS voting procedure is to select the most reliable TIS for each multigene. To do this we implement an approach based on the concept of majority voting. This strategy has also been used to improve genome annotation accuracy in several recent studies [24,31].

In this procedure, for each multigene *m* in each multigene cluster $c\in C_{M}^{*}$, we try to find a TIS (originally annotated or transferred) that belongs to a connected component of the ORF consolidation graph, where the connected component satisfies the following two conditions: (i) it has TISs (originally annotated or transferred) present in at least 80% of the multigenes in *c*; and (ii) it has TISs originally annotated in at least 50% of the multigenes in *c*, or it has TISs originally annotated in at least twice the number of multigenes in *c* than all other connected components in *c*. If such a TIS is found, it is selected as the TIS for *m*. If such a TIS is not found, but *m* has an originally annotated TIS, then the originally annotated TIS is selected as the TIS for *m*. If both of these two cases cannot be applied, the TIS corresponding to the longest ORF in the multigene *m* is selected. After the TIS voting procedure, every multigene has exactly one TIS selected. Thus, we obtain unambiguous TIS annotation for every gene.

Note that the connected components of the sequence consolidation graph—after shared-end edges have been removed— are in a natural one-to-one correspondence with the connected components in the ORF consolidation graph. So in eCAMBer, we implement the TIS voting procedure using the sequence consolidation graph, as it tends to be smaller for closely related genomes.

### Clean up procedure

The last step of eCAMBer is the *clean up procedure*, which is designed to filter out multigene clusters which are likely due to gene annotation errors propagated during the closure procedure.

The input for this procedure consists of the set of multigene clusters $C_{M}^{*}$ and multigene annotations $M_{s}^{c}$, for each strain *s*∈*S*. For each multigene cluster $c\in C_{M}^{*}$ we compute the following features: (i) *l*, the median multigene length in *c*; (ii) *p*, the ratio of the number of strains with at least one element from *c* to the total number of strains; (iii) *r*, the ratio of the number of strains with at least one originally annotated multigene to the total number of strains with at least one element from *c*; (iv) *v*, the ratio of the number of multigenes in the cluster that are overlapped by a longer multigene to the total number of multigenes in the cluster.

Then, we update the set of multigene clusters $C_{M}^{*}$, by removing of multigene clusters for which ($p<\frac{1}{3}$ or $r<\frac{1}{3}$) and (*l*<150 or *v*>0.5).

### Other features

In order to make eCAMBer more user friendly we have added a functionality for downloading genome sequences and genome annotations from the PATRIC database, for the set of selected strains within a species. The downloaded data is automatically formatted as input for eCAMBer. Additionally, eCAMBer integrates Prodigal to generate input gene annotations.

Furthermore, eCAMBer generates output compatible with CAMberVis [27], a tool for simultaneous visualization of multiple genome annotations of bacterial strains. CAMBerVis also handles visualization of genome annotation inconsistencies.

## Results and discussion

In this section we present the results of our experiments, which demonstrate that: (i) eCAMBer is much more efficient than CAMBer, Mugsy-Annotator and the GMV pipeline; (ii) it scales well to large datasets; (iii) it improves annotation consistency; (iv) it improves annotation accuracy; and (v) eCAMBer outperforms Mugsy-Annotator and the GMV pipeline in terms of accuracy.

### Comparison of running times

First, we compare the efficiency of eCAMBer and CAMBer by running the closure procedure for both tools on four datasets from our previous work on CAMBer [11]. All computations in this experiment were performed on the same desktop machine with 4 processor cores being used. In this experiment eCAMBer significantly outperforms CAMBer (Table 1). For example, the running time on 9 strains of *M. tuberculosis* was reduced from about 1 hour 22 minutes to only 42 seconds.

**Table 1:** eCAMBer vs. CAMBer

	**CAMBer**		**eCAMBer**	
**Dataset**	**BLASTs**	**closure**	**BLASTs**	**closure**
2 strains of S. aureus	1 m 47 s	2 m 5 s	8 s	18 s
9 strains of M. tuberculosis	1 h 22 m	1 h 27 m	27 s	41 s
22 strains of S. aureus	6 h	6.5 h	3 m 15 s	4 m
41 strains of E. coli	42 h	48.5 h	22 m	25 m

Second, we also compare the running time of eCAMBer against CAMBer, Mugsy-Annotator and the GMV pipeline by running them on the four datasets from our previous work on CAMBer [11]. Since Mugsy-Annotator does not support multiple thread processing, in this experiment we use only one processor core for the computations. Table 2 presents running times in this experiment. It is clear from this table that the running time speedup achieved by eCAMBer is much more pronounced for larger datasets. This is an expected phenomenon since the other tools have quadratic running times with respect to the number of strains included.

**Table 2:** Comparison of running times for different tools

**Dataset**	**CAMBer**	**eCAMBer**	**Mugsy-Ann.**	**GMV**
2 strains of	7 m 31 s	26 s	2 m	21 m
S. aureus				
9 strains of M.	4 h 12 m	2 m 37 s	1 h 25 m	13 h 53 m
tuberculosis				
22 strains of	37 h 5 m	16 m 30 s	4 h 11 m	28 h 36 m
S. aureus				
41 strains of	273 h 22 m	1 h 48 m	19 h 21 m	368 h 31 m
E. coli				

Comparison of running times between eCAMBer, CAMBer, Mugsy-Annotator and the GMV pipeline on four datasets from our previous work on CAMBer. All computations were executed on a machine with 1 processor core being used. The machine used in this computational experiment was different than the one used in the previous experiment. Columns correspond, in left-to-right order, to: short detaset description, total time consumed by the closure procedure in CAMBer, total time consumed by eCAMBer, total time consumed by Mugsy-Annotator, total time consumed the the GMV pipeline.

The above results also suggest that eCAMBer scales well to larger datasets.

### Large case studies

We examine the scalability of eCAMBer to large datasets by running it on 10 datasets for the 10 species with the highest number of sequenced strains in the PATRIC database [2], in the 16 March 2013 release. All datasets consist of genome sequences and annotations for the sets of strains within the same species. Experiments for all of these datasets were conducted on a machine with 24 processor cores, out of which 20 were used.

Table 3 shows a distribution of running times of all procedures of eCAMBer. The reader may observe that the running times are not necessarily monotonically increasing with the number of strains. For example, the closure procedure computations for the dataset of 162 strains of *H. pylori* took longer than the larger dataset of 195 strains of *S. aureus*. This may be explained by the fact that the total number of distinct sequences for annotated genes in *S. aureus* (98562) is much smaller than in *H. pylori* (208790).

**Table 3:** eCAMBer on large datasets

**Detaset description**				**Running times**				
**Species name**	**Strains**	**Genes**	**Distinct seq.**	**Closure**	**Graph**	**Refine.**	**TIS v.**	**Clean up**
E. coli	569	2923165	487141 (0.17)	12 h	59 m	2 h 51 m	14 m	10 m
S. enterica	293	1366439	244450 (0.18)	3 h 56 m	18 m	36 m	4 m	4 m
S. agalactiae	250	517648	56215 (0.11)	29 m	2 m	5 m	37 s	53 s
S. pneumoniae	238	529076	99578 (0.19)	2 h 29 m	5 m	9 m	1 m 30 s	1 m 10 s
S. aureus	195	523557	98562 (0.19)	1 h 7 m	3 m	4 m	1 m50 s	1 m
H. pylori	163	267302	208790 (0.78)	1 h 42 m	12 m	5 m	5 m 10 s	2 m 10 s
L. interrogans	139	649916	175899 (0.27)	1 h 30 m	4 m	7 m	1 m 30 s	1 m 50 s
V. cholerae	130	467413	97258 (0.21)	24 m	2 m	2 m 20 s	35 s	51 s
A. baumannii	131	487775	129089 (0.27)	34 m	3 m	2 m 30 s	52 s	58 s
B. cereus	104	602986	395477 (0.66)	1 h 13 m	6 m	3 m 50 s	2 m 57 s	1 m 52 s

Running times of eCAMBer on the 10 large datasets. All experiments were performed on the same machine with 24 processor cores, where 20 of them were used. The columns correspond in left-to-right order to: the species name, the number of sequenced strains within the species, the total number of annotated genes, the number of distinct sequences for the set of annotated genes (in the brackets we also provide the ratio between the number of distinct sequences to the total number of annotated genes), running time to compute all BLASTs for the closure procedure, total running time to compute the closure procedure (including BLAST computations), the running time to construct the sequence consolidation graph, the running time to compute the refinement procedure, the running time for the TIS voting procedure, and the running time for the clean up procedure.

In order to further investigate the scalability of eCAMBer, we check how the number of distinct gene sequences increases, when more strains are included. For this experiment, we chose the largest dataset of 569 strains of *E. coli*. Next, we sorted all genomes from the smallest to the largest. The plots (Figure 3) present the number of annotated genes and the number of gene sequences in a cumulative manner. We observe that the total number of distinct sequences grows much slower than the total number of gene annotations, suggesting sub-linear growth of the number of distinct gene sequences. Thus, according to our theoretical considerations, the algorithm implemented in eCAMBer for computing the closure procedure is sub-quadratic with respect to the number of strains included.

This experiment also shows that the strategy applied in eCAMBer to work with unique ORF sequences, rather than ORF annotations, leads to a sequence consolidation graph that is significantly smaller than the corresponding ORF consolidation graph. For example, in the largest dataset for 569 strains of *E. coli*, there is about 12.4mln nodes (ORF annotations) and 2.8bln edges in the ORF consolidation graph, whereas there are only about 1.6mln nodes (unique ORF sequences), 1.3mln shared-end edges, and 55.9mln BLAST-hit edges in the sequence consolidation graph.

### Annotation consistency

We also investigate ability of eCAMBer to identify annotation inconsistencies and to improve the consistency of annotations. As a case study, we use the set of 20 *E. coli* strains with manually curated annotations, deposited in the ColiScope database [5], available through the web-based interface MaGe [32]. Pseudogenes were excluded from the analysis. On this dataset we run the closure procedure, followed by: the refinement procedure, the TIS voting procedure, and the clean up procedure. For comparison we also include annotations for the same set of strains, but downloaded from the PATRIC database [2].

In order to assess the improvement of annotation consistency, after running eCAMBer, we calculated the mean absolute difference in the number of annotated multigenes between two neighbour strains. It is 311 for the original annotations from ColiScope vs. 159 after applying eCAMBer. Analogous statistics on the dataset from PATRIC are 409 for the original annotations and 311 after applying eCAMBer.

In the dataset of 20 *E. coli* strains from ColiScope database, after the closure procedure, eCAMBer identifies 73 gene families which have the following property: each family has a member in every strain, and for each family exactly one strain has a missing original annotation in that family. The top three strains with the highest number of missing gene annotations of that type are: *Sd197* (13), *2a 2457T* (8) and *536* (7). The most well-studied strain *K-12 MG1655* has four missing annotations of the above described type. These annotations were added by eCAMBer during the closure procedure.

Based on this case study, we also investigate how eCAMBer improves consistency of TISs. There are 8038 pairs of originally annotated genes with different TISs, but with identical sequence (including 100bp. upstream region from the TIS of the longer annotation). This number was reduced to 4230 after applying the TIS majority voting procedure and the clean up procedure.

This case study also shows that inconsistencies, which come from annotation errors, are present even for a very well-studied bacterial organism like *E. coli*. Note also that the discussed annotation inconsistencies were identified among strains with annotations curated by the same laboratory.

### Comparison of features of eCAMBer and other tools

CAMBer, eCAMBer, Mugsy-Annotator and the GMV pipeline aim to improve annotation consistency and accuracy. But there are some important differences between these approaches and their features (Table 4). For example, CAMBer and Mugsy-Annotator require gene annotations to be provided, whereas the GMV pipeline generates the input annotations using Prodigal and there is no straightforward way to substitute these annotations with any other. Thus, in all computational experiments involving the GMV pipeline were run only on Prodigal annotations. eCAMBer also integrates Prodigal as a tool to generate input annotations; however, it also allows the user to provide any other annotations as the input. All the tools require genome sequences at the input.

**Table 4:** Qualitative comparison of different tools

	**CAMBer**	**eCAMBer**	**Mugsy-Annotator**	**GMV**
**Input data**	**GS, GA**	**GS, optional GA**	**GS, GA**	**GS**
Mapping of similar sequences	BLAST	BLAST	Multiple WGA	BLAST
Detection of gene presence inconsistencies	Yes	Yes	Yes	No
Detection of gene start inconsistencies	Yes	Yes	Yes	Yes
Correction of gene presence annotations	No	Yes (add. and rem.)	Yes (only add.)	No
Correction of gene start annotations	No	Yes	Yes	Yes
Multithreading	Partial	Yes	No	Partial

Qualitative comparison of different tools. Columns correspond to the tools, whereas rows correpond to different qualitative features of these tools. Acronyms “GS” and “GA” denote genome sequences and genome annotations, respectively. Acronym “WGA” stands for whole genome alignment. Both CAMBer and the GMV pipeline have partial support for multithreading computations since only BLAST computations can be executed in parallel.

Different tools also aim in solving different annotation problems. For example, the GMV pipeline only identifies and solves TIS annotation inconsistencies, whereas Mugsy-Annotator also tries to identify missing genes. Our new tool, eCAMBer, is capable of resolving TIS inconsistencies, as well as removal of overannotated genes and addition of missing genes (Table 4). Our previous tool only identifies annotation inconsistencies, but it does not propose corrections.

Support for multithreading is a valuable feature for computationally demanding problems. Thus, it should be noted that eCAMBer has the most comprehensive support for multithreading among the tools considered. It allows the use of multiple threads for each of its steps. The GMV pipeline and CAMBer support multithreading only for BLAST computations. Mugsy-Annotator does not support it (Table 4).

### Evaluation of annotation accuracy

In order to evaluate accuracy of annotations produced by eCAMBer, Mugsy-Annotator and the GMV pipeline, we apply the tools to annotations produced by the automatic annotation pipeline in PATRIC [2] for the set of 20 *E. coli* strains with manually curated annotations in the ColiScope database [5]. As an alternative dataset of input annotations for the same set of strains we use annotations generated using Prodigal [16].

In all our comparative experiments we run Mugsy-Annotator and the GMV pipeline with default parameters. It should also be mentioned that both Mugsy-Annotator and the GMV pipeline output lists of suggestions of changes to input annotations, rather than actually output the corrected annotations. We post-processed these proposed lists of changes to generate the output annotations used for the comparative experiments.

First we assess the correctness of the changes introduced to the input annotations based on the dataset of gene annotations with experimental support available in the EcoGene 3 database [31]. This dataset consist of 922 gene annotations for the *K-12 MG1655* strain. From this set we excluded four genes: *fdhF*, *prfB*, *rph’*, *insN’*; since their sequences corresponding to the annotated coordinates are disrupted (the length of the sequence from the start codon to the stop codon is not divisible by 3). Additionally, we ran one iteration of the eCAMBer closure procedure to transfer the set of 918 gene annotations on the remaining 19 strains. The transferred gene annotations share at least 80% of sequence identity with original annotations for strain *K-12 MG1655*.

Table 5 presents statistics for the TIS changes introduced by different tools compared against the dataset described above. There are three different scenarios: (i) a correct TIS annotation is changed to an incorrect one (orange); (ii) an incorrect TIS annotation is changed to another incorrect TIS (yellow); (iii) an incorrect TIS is changed to the correct one (green). Since for each gene, there is only one TIS annotation considered as correct, there is no possible change from one correct TIS to another one. For each strain the majority of TIS changes introduced by eCAMBer is correct. In this experiment eCAMBer made 89 TIS changes from incorrect to correct and only 12 TIS changes from correct to incorrect on the dataset of Prodigal annotations. For comparison, GMV made 47 incorrect to correct TIS changes and 8 correct to incorrect TIS changes, on the same dataset. Thus, the number of correct TIS annotations has increased by 77 in case of eCAMBer and by 39 in case of GMV. Application of Mugsy-Annotator made more wrong changes than correct. Additional file 1 shows panel figures for results of eCAMBer, Mugsy-Annotator and GMV on both PATRIC and Prodigal annotations.

**Table 5:** Overall statistics for TIS changes

	**PATRIC**		**Prodigal**		
**Statistic**	**MA**	**eCAMBer**	**GMV**	**MA**	**eCAMBer**
# of incorrect →correct	839	392	47	132	89
TIS changes					
# of incorrect →incorrect	215	50	5	96	8
TIS changes					
# of correct →incorrect	892	92	8	672	12
TIS changes					

Overall statistics for TIS changes introduced by eCAMBer, Mugsy-Annotator (MA) and the GMV pipeline. The tools were run on the dataset of 20 *E. coli* with annotations from the PATRIC database (columns 2 to 3) and generated using Prodigal (columns 4 to 6). Correctness of the changes introduced was assessed by comparison them against the set of experimentally verified gene annotations available in the EcoGene 3 database for the *K-12 MG1655* strain. Gold standard annotations for the remaining 19 strains were obtained by homology transfer of that set of 918 annotations. Statistic presented in this table include only that subset of genes which share the same stop codon as any of the genes in the gold standard.

Since the extended dataset of annotations from Ecogene 3 constitutes only about 20% of all genes in the 20 strains of *E. coli* it is not sufficient for direct assessment of overall quality of changes introduced by eCAMBer and other tools. In particular we cannot conclude if a gene annotation is correct or not based on its absence in this dataset (so that there is no gene annotations in the dataset sharing the same stop codon). Thus, we perform further assessment of the quality of changes introduced relying on manually curated annotations for the set of 20 *E. coli* strains in the ColiScope dataset [5]. It is a reasonable choice as a gold standard, since many of the annotations have experimental support. In particular, the annotation for the strain *K-12 MG1655* contains 901 out of 918 gene annotations present in the dataset described previously. For comparison, for this strain, there are only 841 and 883 such gene annotations for PATRIC and Prodigal, respectively.

**Figure 5:**
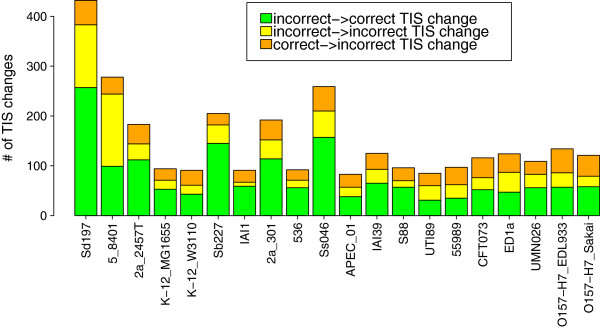
**Statistics for TIS voting procedure.** Impact of the TIS voting procedure of eCAMBer on annotations from the PATRIC database. Annotations from the ColiScope database were used to assess correctness of TIS changes. Note, that since for each gene, there is only one TIS annotation considered as correct, thus there is no possible change from one correct TIS to another one.

Next, Figure 5 presents the assessment of TIS changes introduced during the TIS voting procedure based on the ColiScope dataset. It shows the assessment of the TIS changes introduced to the input PATRIC annotations, with respect to each of the 20 *E. coli* strains. Statistic presented in this figure distinguishes three different scenarios: (i) a correct TIS annotation is changed to an incorrect one (orange); (ii) an incorrect TIS annotation is changed to another incorrect TIS (yellow); (iii) an incorrect TIS is changed to the correct one (green). Since for each gene, there is only one TIS annotation considered as correct, there is no possible change from one correct TIS to another one. For each strain the majority of TIS changes introduced by eCAMBer is correct. Additional file 2 shows analogous panel figures for results of eCAMBer, Mugsy-Annotator and GMV on both PATRIC and Prodigal annotations. Rows 5 to 8 of Table 6 summarize the overall impact of eCAMBer and Mugsy-Annotator on TIS annotations. Remarkably, 70% (1591 out of 2260) of TIS changes introduced by eCAMBer to PATRIC annotations were correct. For comparison, only 43% of the TIS changes introduced by Mugsy-Annotator were correct.

**Table 6:** Overall accuracy statistics for different tools

	**PATRIC**			**Prodigal**			
**Statistic**	**Input**	**MA**	**eCAMBer**	**Input**	**GMV**	**MA**	**eCAMBer**
# of incorrectly removed genes	NA	0	1224	NA	0	0	388
# of incorrectly added genes	NA	1177	792	NA	0	344	331
# of correctly removed genes	NA	0	3993	NA	0	0	1185
# of correctly added genes	NA	410	701	NA	0	210	1447
# of incorrect →correct TIS changes	NA	4812	1591	NA	149	1015	290
# of incorrect →incorrect TIS changes	NA	2223	747	NA	28	1018	113
# of correct →incorrect TIS changes	NA	4279	669	NA	78	3618	170
Precision for gene starts	0.665	0.663	0.699	0.764	0.764	0.734	0.775
Recall for gene starts	0.695	0.702	0.703	0.752	0.753	0.727	0.765
f1 for gene starts	0.680	0.682	0.701	0.758	0.759	0.731	0.770
Precision for gene ends	0.892	0.882	0.920	0.931	0.931	0.928	0.940
Recall for gene ends	0.931	0.935	0.926	0.917	0.917	0.919	0.927
f1 for gene ends	0.911	0.908	0.923	0.924	0.924	0.923	0.934

Overall statistics for accuracy of changes introduced by eCAMBer, Mugsy-Annotator (MA) and the GMV pipeline. The tools were run on the dataset of 20 *E. coli* with annotations from the PATRIC database (columns 2 to 4) and generated using Prodigal (columns 5 to 8). Correctness of the changes introduced was assessed by comparison with annotations from the Coliscope database. Columns Input correspond to the original annotations. “NA” stands for not applicable. Rows correspond to different statistics of running each tool.

**Figure 6:**
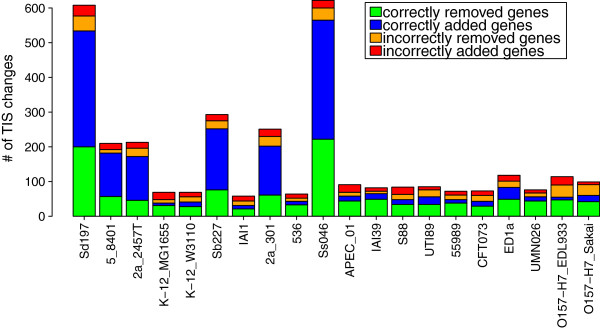
**Statistics for closure and clean up procedures.** Impact of the closure and clean up procedures of eCAMBer on the annotations from the PATRIC database. Annotations from the ColiScope database were used to assess correctness of gene removals and additions introduced by eCAMBer.

Figure 6 presents the assessment of gene additions and removals introduced during the closure and the clean up procedures, respectively. It shows the assessment of the changes introduced to the input PATRIC annotations, with respect to each of the 20 *E. coli* strains. Statistic presented in this figure distinguishes four different scenarios: (i) a missing genome annotation is correctly added during the closure procedure (blue); (ii) a wrong gene annotation is correctly removed during the clean up procedure (green); (iii) a wrong gene annotation is incorrectly added during the closure procedure (red); and (iv) a correct gene annotation is incorrectly removed during the clean up procedure (orange). It can be seen that, for each strain, the majority of changes introduced by eCAMBer is correct. Additional file 3 shows analogous panel figures for results of Mugsy-Annotator and eCAMBer on both PATRIC and Prodigal annotations. The first four rows of Table 6 summarize the overall impact of eCAMBer and Mugsy-Annotator on gene presence. The results show that eCAMBer outperforms Mugsy-Annotator in this aspect. For example, 70% of the changes introduced by eCAMBer to PATRIC annotations were correct, whereas it was only 26% for Mugsy-Annotator.

Finally, we investigate how the whole pipelines implemented in eCAMBer, Mugsy-Annotator and GMV improve the overall annotation accuracy. Here, the accuracy is measured by *f*_1_ statistic, defined as $2\cdot \frac{\text{precision}\cdot \text{recall}}{\text{precision}+\text{recall}}$, where $\text{precision}=\frac{\text{TP}}{\text{TP}+\text{FP}}$ and $\text{recall}=\frac{\text{TP}}{\text{TP}+\text{FN}}$. Here, *TP*, *FP* and *FN* denote true positive, false positive and false negative prediction, respectively. Since a pair of gene annotations may have the same stop codon, but different TISs, we keep track on the results for both stop codon predictions and for the TIS predictions.

**Figure 7:**
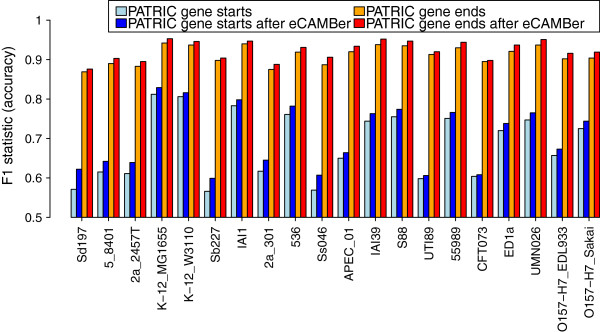
**Comparison of annotation accuracy before and after applying eCAMBer.** Comparison of annotation accuracy before and after applying eCAMBer on the dataset of 20 *E. coli* strains with annotations from PATRIC. Manually curated annotations from ColiScope were used as a gold standard.

Results of eCAMBer on PATRIC annotations in this experiment are presented in Figure 7. Note that each correctly identified TIS determines also its correctly identified stop codon, but not the other way round. Thus, the accuracy for the TIS prediction is lower than for the stop codons. As the figure shows, eCAMBer improves annotation accuracy, for each strain, both in terms of TIS annotations and stop codon annotations. Additional file 4 shows analogous panel figures for results of eCAMBer, Mugsy-Annotator and GMV on both PATRIC and Prodigal annotations. Rows 9 and 12 of Table 6 summarize the change in accuracy when running different tools on PATRIC and Prodigal annotations. It is clear from this table that eCAMBer outperforms other tools. For example, eCAMBer increased the f1 statistic of initial annotations of Prodigal (for gene starts) from 0.764 to 0.775, whereas the application of GMV improved it only by 0.001 and the application of Mugsy-Annotator decreased it by 0.027. In the case of PATRIC annotations, application of Mugsy-Annotator improved the accuracy from 0.680 to 0.682. However, the accuracy of annotations after eCAMBer increased to 0.703.

## Conclusions

We have developed eCAMBer, a tool for supporting large-scale comparative analysis of multiple bacterial strains. eCAMBer identifies and resolves annotation inconsistencies among closely related bacterial genomes.

This tool works in two phases. First, it tries to transfer gene annotations among all considered bacterial strains. In this procedure, called closure, it also identifies homologous gene families and annotation inconsistencies. The underlying idea behind the efficient implementation of the procedure is to avoid redundant BLAST queries. This approach greatly reduces the computational complexity, thus leading to much shorter running time than other tools. For example, on the dataset of 41 strains of *E. coli*, computations took less than two hours (using only one processing thread), whereas Mugsy-Annotator (the fastest competitor) took more than 19 hours. Moreover, eCAMBer supports multithreading for all its procedures. This allows eCAMBer to be used on much larger datasets comprising hundreds of bacterial strains.

An idea, called compressive genomics, has recently been proposed with new approaches to optimize BLAST search time of sequence databases [33,34]. However, one significant conceptual difference, between these methods and the closure procedure in eCAMBer, is that these approaches try to reduce the size of the target database, whereas the eCAMBer closure procedure reduces the redundancy among BLAST queries. It may be interesting, for further research, to combine these ideas.

In the second phase, eCAMBer applies a majority voting-like approach, in the procedure called TIS voting, to choose the most reliable TIS for each gene. Finally, it removes possible gene annotation errors during the clean up procedure. Our case study experiments show that, in these steps, eCAMBer improves the quality of initial annotations generated with automatic pipelines, such as PATRIC or Prodigal. For example, the application of eCAMBer to PATRIC annotations performed 1575 TIS changes, out of which 1183 (75%) were correct.

Moreover, eCAMBer outperforms its competitors, Mugsy-Annotator and the GMV pipeline, in terms of improving quality of annotations. In particular, when run on Prodigal annotations for the set of 20 *E. coli* strains, eCAMBer increased the f1 statistic of initial annotations from 0.764 to 0.775, whereas the application of GMV improved it only by 0.001 and the application of Mugsy-Annotator even decreased it.

Finally, eCAMBer also has some limitations. One is that it purely relies on the quality of original annotations. Thus, for example, eCAMBer cannot identify genes, whose annotations are missing for all strains. Another limitation of eCAMBer is that pseudogenes and non-protein coding genes are excluded from the analysis. This follows from the assumption that eCAMBer considers only genes that start with start codon, end with stop codon, and have length divisible by 3.

## Competing interests

The authors declare that they have no competing interests.

## Authors’ contributions

All authors contributed to design of the method, analysis of results and writing of the manuscript. MW wrote software and performed experiments. All authors read and approved the final manuscript.

## Supplementary Material

Additional file 1 **Assessment of the correctness of TIS changes based on Ecogene 3.0.** Comparison of the impact of applying eCAMBer, Mugsy-Annotator and the GMV pipeline on the quality of TIS annotations. The experiment was run on the dataset of 20 *E. coli* strains with annotations downloaded from PATRIC and generated using Prodigal. Correctness of changes introduced was assessed by comparison with the set of annotations downloaded from the EcoGene 3 database for the *K-12 MG1655* strain plus transferred annotations for the 19 remaining strains. Click here for file

Additional file 2 **Assessment of the correctness of TIS changes based on ColiScope.** Comparison of the impact of applying eCAMBer, Mugsy-Annotator and the GMV pipeline on the quality of TIS annotations. The experiment was run on the dataset of 20 *E. coli* strains with annotations downloaded from PATRIC and generated using Prodigal. Correctness of changes introduced was assessed by comparison with annotations in the ColiScope database. Click here for file

Additional file 3 **Assessment of the correctness of gene removals and additions.** Comparison of the impact of applying eCAMBer, Mugsy-Annotator and the GMV pipeline on the quality of gene ends annotations. The experiment was run on the dataset of 20 *E. coli* strains with annotations downloaded from PATRIC and generated using Prodigal. Correctness of changes introduced was assessed by comparison with annotations in the ColiScope database. Click here for file

Additional file 4 **Accuracy: eCAMBer vs. other tools.** Comparison of the impact of applying eCAMBer, Mugsy-Annotator and the GMV pipeline on accuracy annotations. To asses the accuracy *f*_1_ statistic was used. The experiment was run on the dataset of 20 *E. coli* strains with annotations downloaded from PATRIC and generated using Prodigal. Correctness of changes introduced was assessed by comparison with annotations in the ColiScope database. Click here for file
