# Supplementary figures and images for: eCAMBer: efficient support for large-scale comparative analysis of multiple bacterial strains
Source: BMC Bioinformatics. 2014 Mar 5;15:65. doi: 10.1186/1471-2105-15-65 (PMC4023553; doi:10.1186/1471-2105-15-65)

# Assessment of the correctness of TIS changes based on Ecogene 3.0

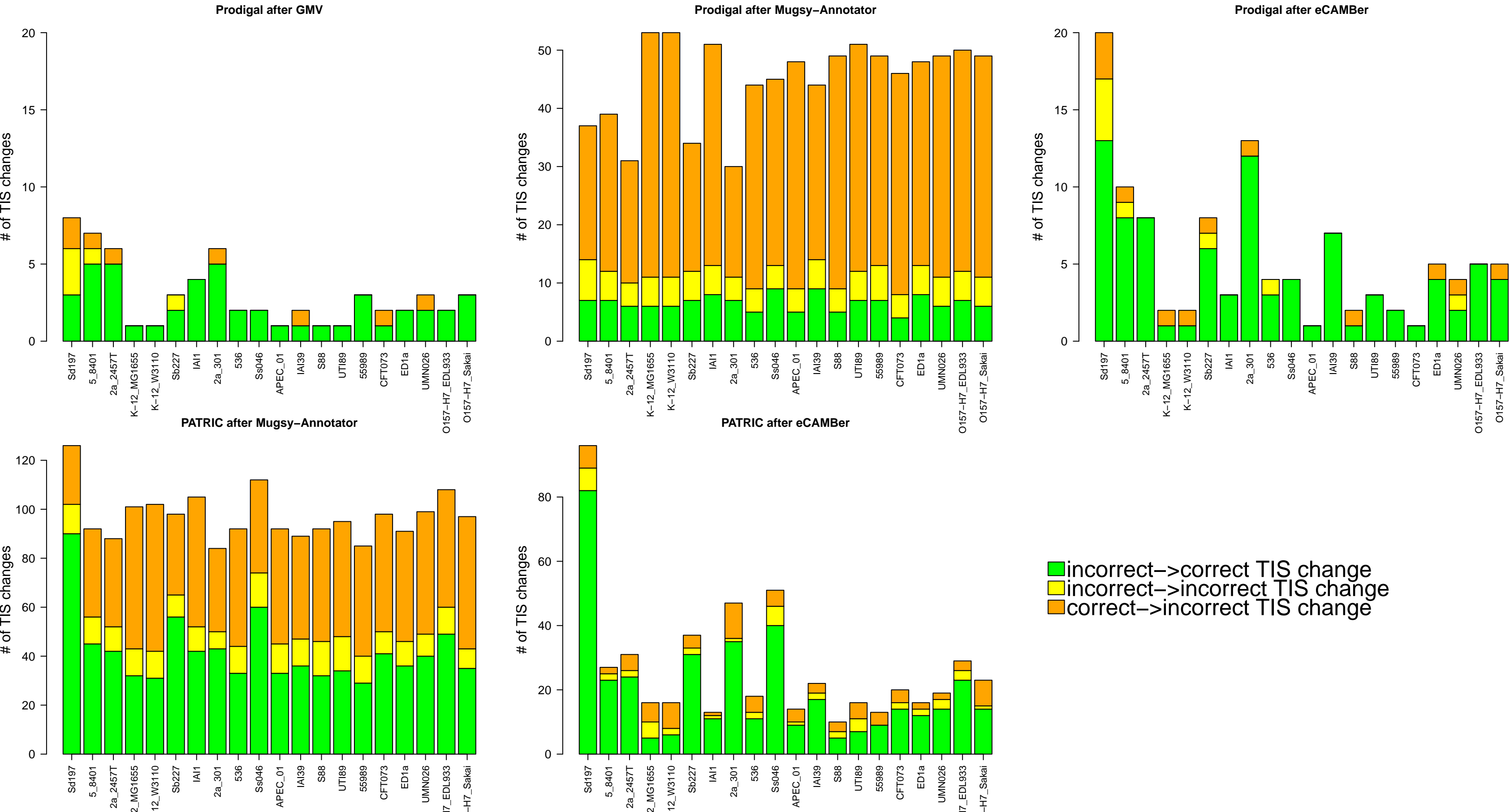

Supplement: Additional file 1 — Assessment of the correctness of TIS changes based on Ecogene 3.0. Comparison of the impact of applying eCAMBer, Mugsy-Annotator and the GMV pipeline on the quality of TIS annotations. The experiment was run on the dataset of 20 E. coli strains with annotations downloaded from PATRIC and generated using Prodigal. Correctness of changes introduced was assessed by comparison with the set of annotations downloaded from the EcoGene 3 database for the K-12 MG1655 strain plus transferred annotations for the 19 remaining strains. [file 1471-2105-15-65-S1.pdf]

# Assessment of the correctness of TIS changes based on ColiScope

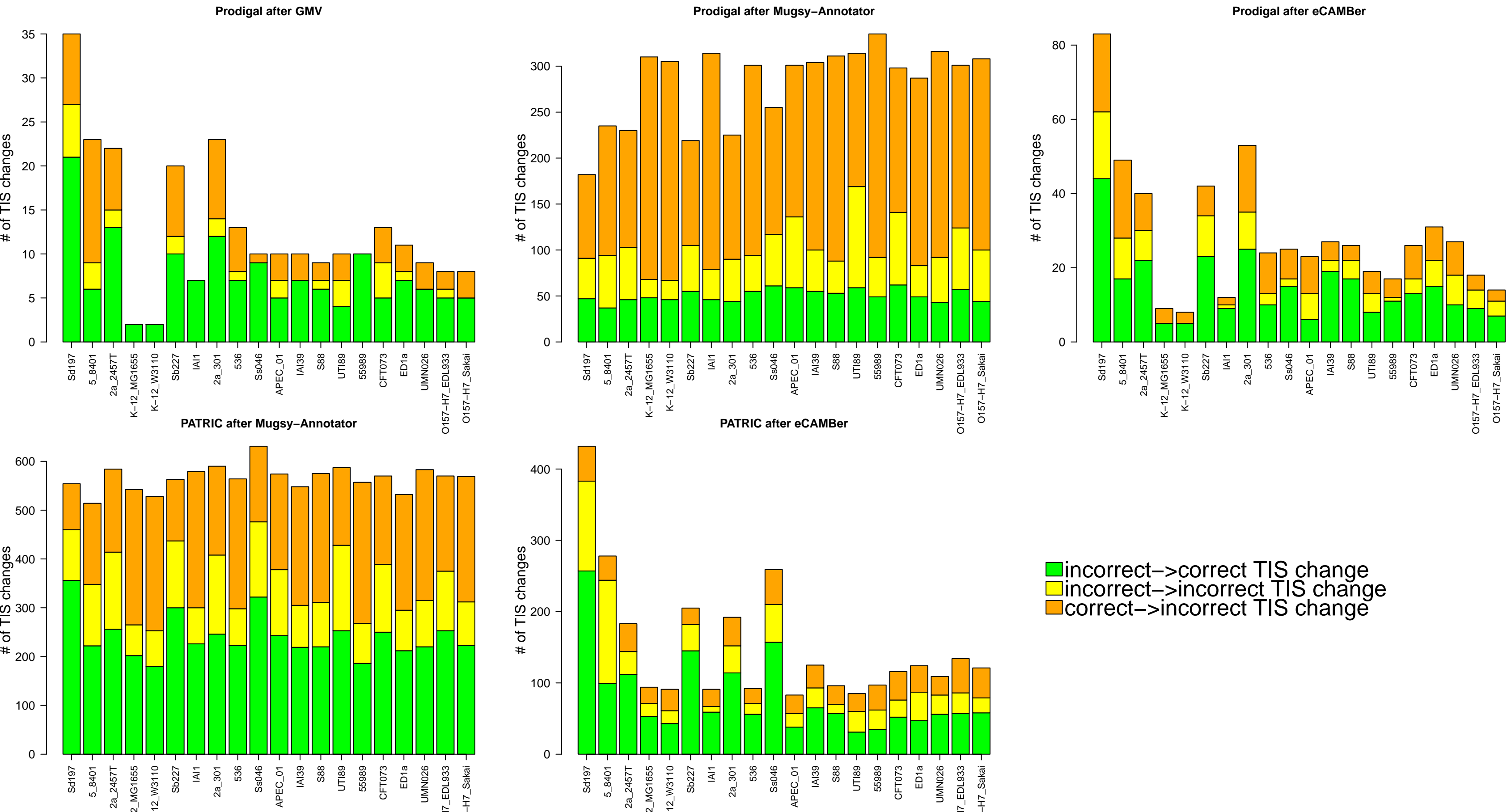

Supplement: Additional file 2 — Assessment of the correctness of TIS changes based on ColiScope. Comparison of the impact of applying eCAMBer, Mugsy-Annotator and the GMV pipeline on the quality of TIS annotations. The experiment was run on the dataset of 20 E. coli strains with annotations downloaded from PATRIC and generated using Prodigal. Correctness of changes introduced was assessed by comparison with annotations in the ColiScope database. [file 1471-2105-15-65-S2.pdf]

# Accuracy: eCAMBer vs. other tools.

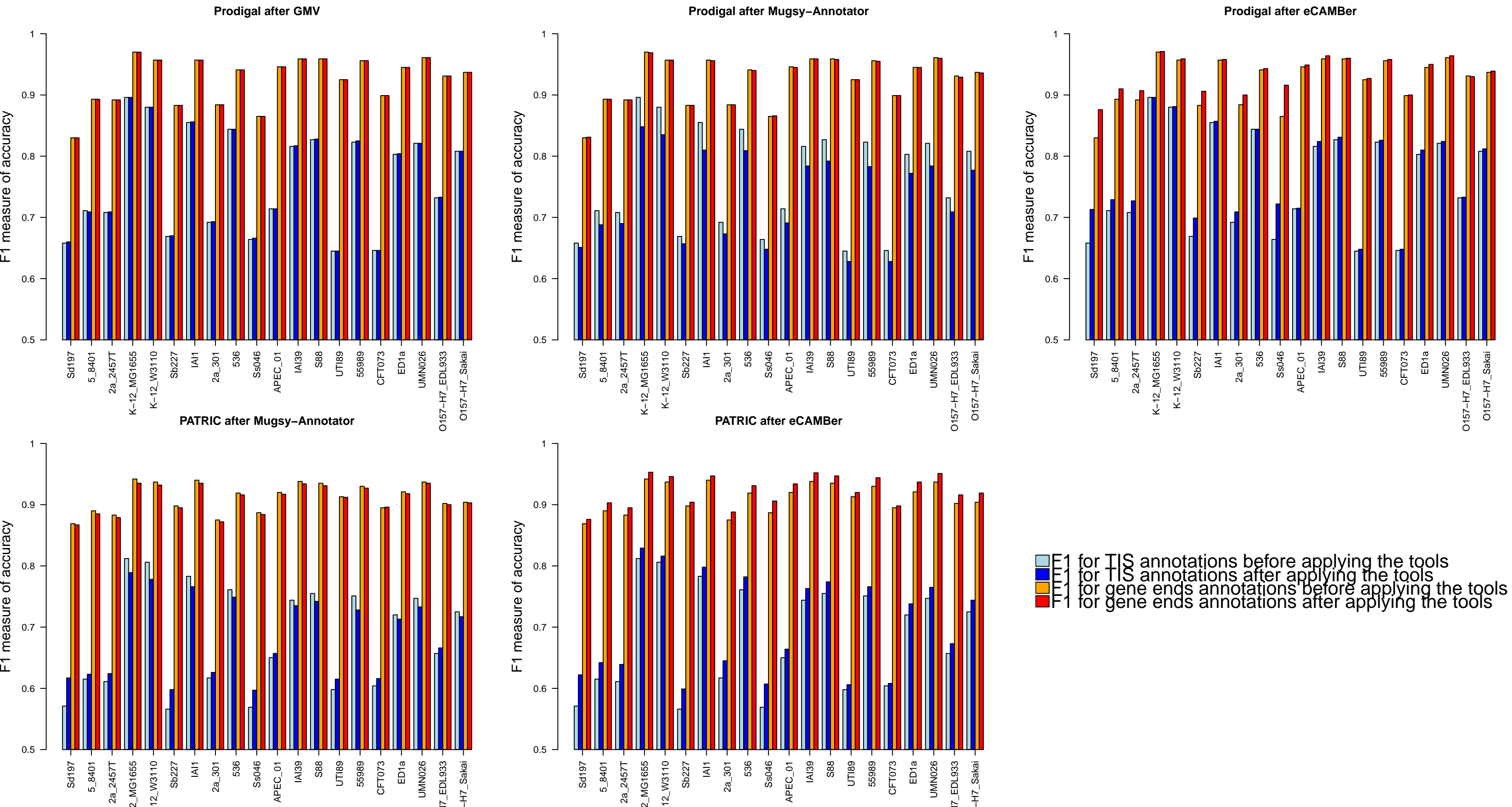

Supplement: Additional file 4 — Accuracy: eCAMBer vs. other tools. Comparison of the impact of applying eCAMBer, Mugsy-Annotator and the GMV pipeline on accuracy annotations. To asses the accuracy f1 statistic was used. The experiment was run on the dataset of 20 E. coli strains with annotations downloaded from PATRIC and generated using Prodigal. Correctness of changes introduced was assessed by comparison with annotations in the ColiScope database. [file 1471-2105-15-65-S4.pdf]
