# Supplementary material for: eCAMBer: efficient support for large-scale comparative analysis of multiple bacterial strains
Source: BMC Bioinformatics. 2014 Mar 5;15:65. doi: 10.1186/1471-2105-15-65 (PMC4023553; doi:10.1186/1471-2105-15-65)

Assessment of the correctness of gene removals and additions

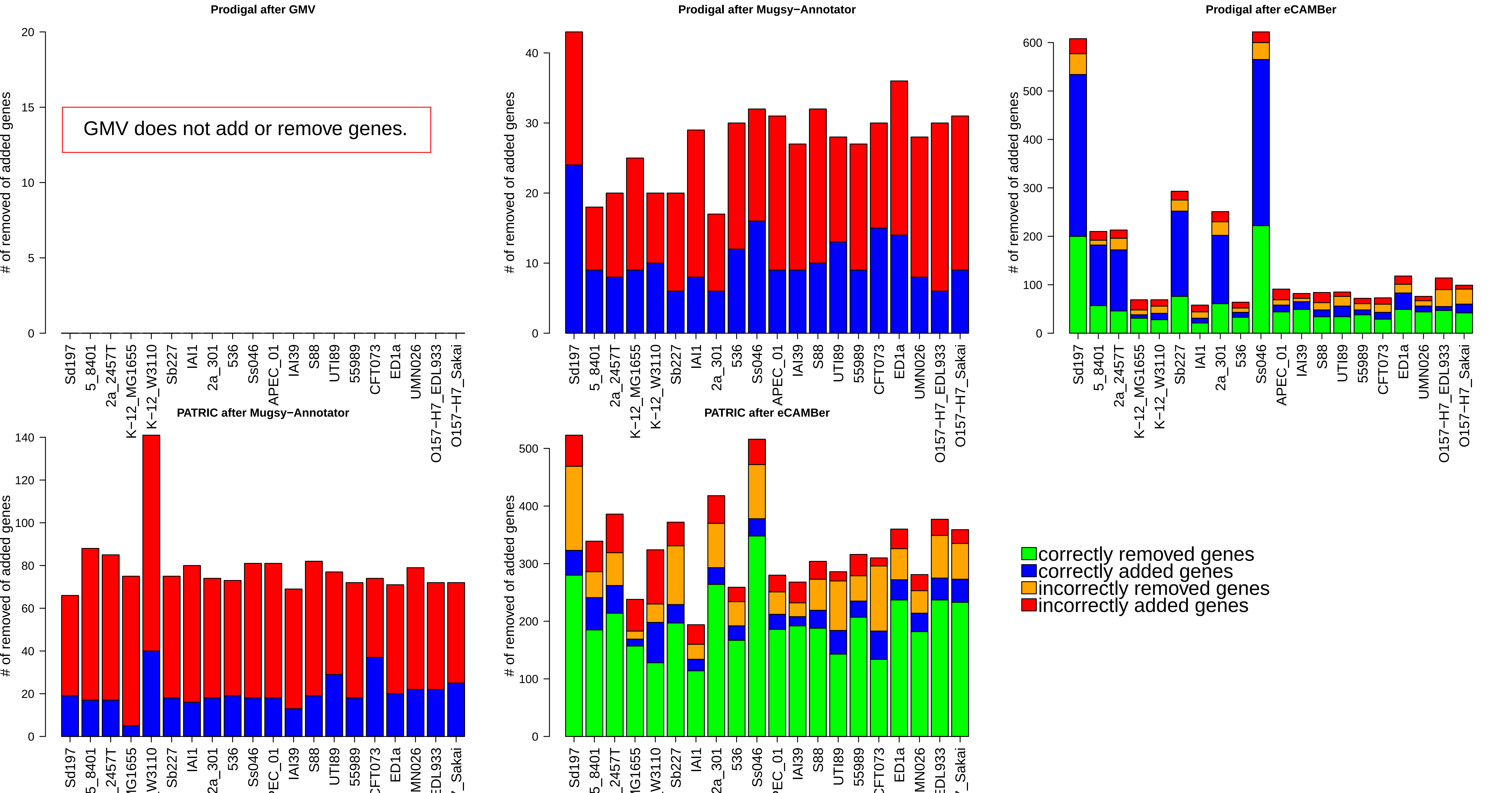

Supplement: Additional file 3 — Assessment of the correctness of gene removals and additions. Comparison of the impact of applying eCAMBer, Mugsy-Annotator and the GMV pipeline on the quality of gene ends annotations. The experiment was run on the dataset of 20 E. coli strains with annotations downloaded from PATRIC and generated using Prodigal. Correctness of changes introduced was assessed by comparison with annotations in the ColiScope database. [file 1471-2105-15-65-S3.pdf]
